# Baseline risk factors of in-hospital mortality after surgery for acute type A aortic dissection: an ERTAAD study

**DOI:** 10.3389/fcvm.2023.1307935

**Published:** 2024-01-15

**Authors:** Fausto Biancari, Till Demal, Francesco Nappi, Francesco Onorati, Alessandra Francica, Sven Peterss, Joscha Buech, Antonio Fiore, Thierry Folliguet, Andrea Perrotti, Amélie Hervé, Lenard Conradi, Andreas Rukosujew, Angel G. Pinto, Javier Rodriguez Lega, Marek Pol, Jan Rocek, Petr Kacer, Konrad Wisniewski, Enzo Mazzaro, Igor Vendramin, Daniela Piani, Luisa Ferrante, Mauro Rinaldi, Eduard Quintana, Robert Pruna-Guillen, Sebastien Gerelli, Dario Di Perna, Metesh Acharya, Giovanni Mariscalco, Mark Field, Manoj Kuduvalli, Matteo Pettinari, Stefano Rosato, Paola D’Errigo, Mikko Jormalainen, Caius Mustonen, Timo Mäkikallio, Angelo M. Dell’Aquila, Tatu Juvonen, Giuseppe Gatti

**Affiliations:** ^1^ Department of Medicine, South-Karelia Central Hospital, University of Helsinki, Lappeenranta, Finland; ^2^ Heart and Lung Center, Helsinki University Hospital, University of Helsinki, Helsinki, Finland; ^3^ Department of Cardiovascular Surgery, University Heart and Vascular Center Hamburg, Hamburg, Germany; ^4^ Department of Cardiac Surgery, Centre Cardiologique du Nord de Saint-Denis, Paris, France; ^5^ Division of Cardiac Surgery, University of Verona Medical School, Verona, Italy; ^6^ LMU University Hospital, Ludwig Maximilian University, Munich, Germany; ^7^ German Centre for Cardiovascular Research, Partner Site Munich Heart Alliance, Munich, Germany; ^8^ Department of Cardiac Surgery, Hôpitaux Universitaires Henri Mondor, Assistance Publique-Hôpitaux de Paris, Creteil, France; ^9^ Department of Thoracic and Cardiovascular Surgery, University of Franche-Comte, Besancon, France; ^10^ Department of Cardiothoracic Surgery, University Hospital Muenster, Muenster, Germany; ^11^ Cardiovascular Surgery Department, University Hospital Gregorio Marañón, Madrid, Spain; ^12^ Department of Cardiac Surgery, Third Faculty of Medicine, Charles University and University Hospital Kralovske Vinohrady, Prague, Czech Republic; ^13^ Division of Cardiac Surgery, Cardio-Thoracic and Vascular Department, Azienda Sanitaria Universitaria Giuliano Isontina, Trieste, Italy; ^14^ Cardiothoracic Department, University Hospital, Udine, Italy; ^15^ Cardiac Surgery, Molinette Hospital, University of Turin, Turin, Italy; ^16^ Department of Cardiovascular Surgery, Hospital Clínic de Barcelona, University of Barcelona, Barcelona, Spain; ^17^ Department of Cardiac Surgery, Centre Hospitalier Annecy Genevois, Annecy, France; ^18^ Department of Cardiac Surgery, Glenfield Hospital, Leicester, United Kingdom; ^19^ Liverpool Centre for Cardiovascular Sciences, Liverpool Heart and Chest Hospital, Liverpool, United Kingdom; ^20^ Department of Cardiac Surgery, Ziekenhuis Oost Limburg, Genk, Belgium; ^21^ National Center for Global Health, Istituto Superiore di Sanitá, Rome, Italy; ^22^ Department of Cardiac Surgery, Martin Luther University Halle-Wittenberg, Halle, Germany; ^23^ Research Unit of Surgery, Anesthesia and Critical Care, University of Oulu, Oulu, Finland

**Keywords:** type A aortic dissection, aortic dissection, TAAD, thoracic aortic aneurysm and dissection, risk prediction, risk factor, arterial lactate

## Abstract

**Background:**

Surgery for type A aortic dissection (TAAD) is associated with high risk of mortality. Current risk scoring methods have a limited predictive accuracy.

**Methods:**

Subjects were patients who underwent surgery for acute TAAD at 18 European centers of cardiac surgery from the European Registry of Type A Aortic Dissection (ERTAAD).

**Results:**

Out of 3,902 patients included in the ERTAAD, 2,477 fulfilled the inclusion criteria. In the validation dataset (2,229 patients), the rate of in-hospital mortality was 18.4%. The rate of composite outcome (in-hospital death, stroke/global ischemia, dialysis, and/or acute heart failure) was 41.2%, and 10-year mortality rate was 47.0%. Logistic regression identified the following patient-related variables associated with an increased risk of in-hospital mortality [area under the curve (AUC), 0.755, 95% confidence interval (CI), 0.729–0.780; Brier score 0.128]: age; estimated glomerular filtration rate; arterial lactate; iatrogenic dissection; left ventricular ejection fraction ≤50%; invasive mechanical ventilation; cardiopulmonary resuscitation immediately before surgery; and cerebral, mesenteric, and peripheral malperfusion. The estimated risk score was associated with an increased risk of composite outcome (AUC, 0.689, 95% CI, 0.667–0.711) and of late mortality [hazard ratio (HR), 1.035, 95% CI, 1.031–1.038; Harrell's C 0.702; Somer's D 0.403]. In the validation dataset (248 patients), the in-hospital mortality rate was 16.1%, the composite outcome rate was 41.5%, and the 10-year mortality rate was 49.1%. The estimated risk score was predictive of in-hospital mortality (AUC, 0.703, 95% CI, 0.613–0.793; Brier score 0.121; slope 0.905) and of composite outcome (AUC, 0.682, 95% CI, 0.614–0.749). The estimated risk score was predictive of late mortality (HR, 1.035, 95% CI, 1.031–1.038; Harrell's C 0.702; Somer's D 0.403), also when hospital deaths were excluded from the analysis (HR, 1.024, 95% CI, 1.018–1.031; Harrell's C 0.630; Somer's D 0.261).

**Conclusions:**

The present analysis identified several baseline clinical risk factors, along with preoperative estimated glomerular filtration rate and arterial lactate, which are predictive of in-hospital mortality and major postoperative adverse events after surgical repair of acute TAAD. These risk factors may be valuable components for risk adjustment in the evaluation of surgical and anesthesiological strategies aiming to improve the results of surgery for TAAD.

**Clinical Trial Registration:**

https://clinicaltrials.gov, identifier NCT04831073.

## Introduction

Type A aortic dissection (TAAD) is an emergency condition that requires prompt surgical repair to reduce the risk of mortality (1). In Western countries, large series demonstrated that early postoperative mortality in these patients is about 17% (2, 3). Early mortality rates lower than 15% have been reported in Asian centers (4–6). Furthermore, surgery for TAAD is often complicated by end-organ injury, which may impact patient recovery. Recently, the UK National Adult Cardiac Surgical Audit (2) and the German Registry of Acute Aortic Dissection Type A (GERAADA) (7) investigators developed two risk scoring methods to predict early postoperative mortality of these patients. The discriminative ability of the regression models of both studies was moderate [area under (AUC) the receiver operating characteristics curve (ROC): 0.694 and 0.725, respectively] (2, 4). A few studies demonstrated that even the European System for Cardiac Operative Risk Evaluation II had a better discriminative ability compared to the GERAADA score (7–9). These observations suggest that other confounding factors, such as biomarkers of tissue perfusion and renal insufficiency, may account for the current limited ability to predict adverse events after surgery for acute TAAD. Undoubtedly, it is not justified to turn down a patient because surgery is too risky based only on the estimation of a risk score. However, the development of a specific risk stratification tool is of utmost importance in clinical research for the evaluation of treatment strategies aiming to reduce mortality after surgery for TAAD. In this regard, identification of only baseline risk factors may be useful for risk adjustment of the effect of different treatment strategies in these patients (7) The present study was performed to identify preoperative patient-level risk factors of prognostic importance from a multicenter TAAD registry.

## Patients and methods

### Study population

The European Registry of Type A Aortic Dissection (ERTAAD) (10) is a retrospective study on consecutive patients operated for acute TAAD at 18 centers of cardiac surgery in eight European countries (Belgium, Czech Republic, Finland, France, Germany, Italy, Spain, and the United Kingdom) from January 2005 to March 2021. Data on pre-specified baseline, operative, and early postoperative outcome variables were collected into a Microsoft Access datasheet (Redmond, WA, USA).

The inclusion criteria of the ERTAAD registry were the following: (1) patients with acute TAAD; (2) patients >18 years old; (3) onset of symptoms within 7 days prior to surgery; (4) primary surgical repair of acute TAAD; (5) any other major cardiac surgical procedure concomitant with surgery for TAAD (9). The exclusion criteria were the following: (1) patients aged <18 years; (2) onset of symptoms more than 7 days prior to surgery; (3) prior procedure for TAAD; (4) retrograde TAAD; (5) concomitant endocarditis; (6) TAAD secondary to blunt or penetrating chest trauma (10).

**Figure 1:**
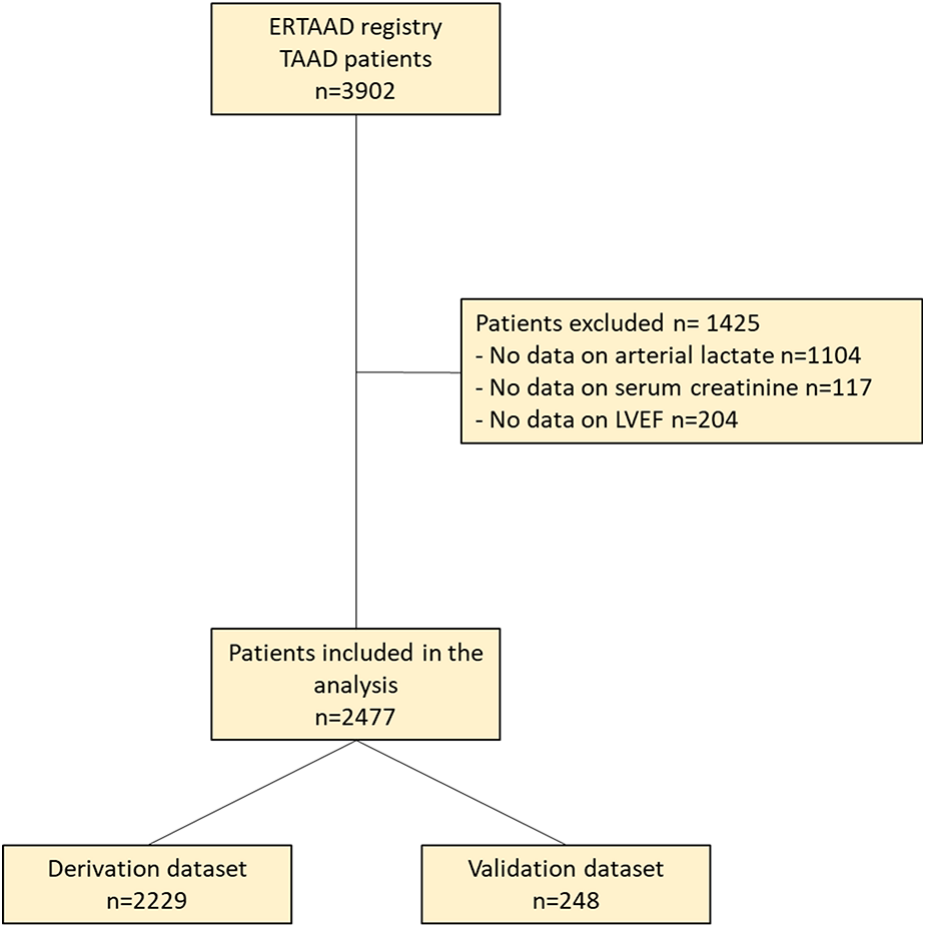
Study flowchart.

For the present study, patients with missing data on preoperative estimated glomerular filtration rate (eGFR), arterial lactate, and left ventricular ejection fraction were excluded from this analysis (Figure 1).

The definition criteria of clinical and operative variables of this study have been previously reported (10). eGFR was calculated according to the Chronic Kidney Disease Epidemiology Collaboration (CKD-EPI) equation (11). Arterial lactate and serum creatinine levels were measured immediately before surgery. Cardiopulmonary resuscitation refers to external chest cardiac massage en route to the operating room or external/open chest cardiac massage immediately after anesthesia induction (10).

### Study outcomes

The primary outcome of this study was in-hospital mortality. Secondary postoperative outcomes were a composite outcome including any of the following major postoperative complications: in-hospital death, stroke/global brain ischemia, dialysis, and acute heart failure occurring during the index hospitalization as well as each of these adverse events. Late mortality was also a secondary outcome of the present analysis.

In-hospital mortality refers to all-cause death occurring during the index hospitalization. Late mortality refers to all-cause death occurring during the index hospitalization or later. Stroke refers to a focal or global neurological deficit with at least one of the following features: change in the level of consciousness, hemiplegia, hemiparesis, numbness, or sensory loss affecting one side of the body, dysphasia or aphasia, hemianopia, amaurosis fugax, or other neurological signs or symptoms consistent with stroke duration of a focal or global neurological deficit ≥24 h or <24 h if available neuroimaging documented a new brain hemorrhage or infarct. Global brain ischemia refers to diffuse hypoxic damage diagnosed at brain imaging and/or electroencephalography. Dialysis refers to any postoperative temporary or permanent renal replacement therapy during the index hospitalization. Postoperative heart failure refers to prolonged use of inotropes (>24 h) and/or the insertion of any mechanical circulatory support device during the index hospitalization (10).

### Statistical analysis

Continuous variables were reported as means and standard deviations as well as medians and interquartile ranges. Categorical variables were reported as counts and percentages. Risk estimates were reported as odds ratios (ORs) and hazard ratios (HRs) with 95% confidence interval (CI). Chi-square, Fisher's exact, and linear-by-linear association tests were used to analyze differences between categorical variables. The Mann–Whitney test was used to compare continuous variables. After assigning a random number to each patient, the dataset was split into a derivation dataset (90% of patients) and a validation dataset (10% of patients). The size of the validation dataset was chosen according to the typical volume of studies investigating institutional series, which is most often less than 300 cases. Indeed, validation of the prognostic accuracy of the estimated risk score is expected to be assessed in datasets of limited size. Logistic regression with the backward stepwise method (probability for stepwise: entry, 0.05; removal, 0.10) included the following baseline variables with *p* < 0.05 in univariable analysis: age, preoperative eGFR, preoperative arterial lactate, bicuspid aortic valve, iatrogenic TAAD, diabetes, extracardiac arteriopathy, left ventricular ejection fraction ≤50%, use of inotropes, invasive mechanical ventilation, cardiopulmonary resuscitation en route to operating room or after anesthesia induction, cerebral malperfusion, renal malperfusion, mesenteric malperfusion, and peripheral malperfusion. No operative variable was included into this regression model because the estimated risk score should be useful to adjust the risk in the evaluation of different treatment strategies. Discrimination of the logistic regression model was assessed by calculating the AUC ROC curve and its calibration with the Hosmer–Lemeshow's test. The Brier score was calculated to evaluate the disagreement between the observed and predicted in-hospital mortality rates. A Brier score should be as close to 0 as possible, with 0.25 as an acceptable upper cutoff. The predictive ability of the estimated probability of in-hospital mortality was assessed in the validation dataset. We aimed to verify the goodness-of-fit of the findings of regression analysis of the derivation dataset, by performing k-fold cross-validation of the logistic regression probabilities considering the entire study cohort. Using the “crossfold” and the “cv_kfold” commands for Stata, we estimated the root mean squared error, the pseudo-R^2^, the mean absolute errors, and the mean log-likelihood in fivefold.

The predictive performance of the risk score in predicting late mortality was evaluated using the Cox proportional hazard method, and concordance between late mortality and prediction was evaluated by calculating Somer's D rank correlation, Harrell's C concordance coefficient, and the Brier score. The Kaplan–Meier method was used to evaluate the performance of quintiles of the risk score in predicting the late mortality. Separate survival analyses were performed in the overall series and in those patients who survived to discharge. Statistical analyses were performed with the SPSS (version 27.0, SPSS Inc., IBM, Chicago, IL, USA) and Stata (version 15.1, StataCorp LLC, College Station, TX, USA) statistical software.

### Ethical statement

The Ethical Review Board of the Helsinki University Hospital, Finland (21 April 2021, Diary No. HUS/237/2021) and the Ethical Review Board of each participating hospital approved this study. The requirement for informed consent was waived because of the retrospective nature of this study.

## Results

### Study population

A total of 3,902 consecutive patients were included in the ERTAAD registry, and 2,477 fulfilled the inclusion criteria of the present analysis (Figure 1). The baseline characteristics and operative data of patients of the derivation and validation datasets are summarized in Table 1. In this series, in-hospital mortality rate was 18.2%, stroke/global brain ischemia rate was 18.5%, dialysis rate was 15.6%, and acute heart failure rate was 16.2%. The rate of composite outcome was 41.2%. Ten-year mortality rate was 47.2%.

**Table 1:** Patients’ characteristics and operative data of patients in the derivation and validation datasets.

	Derivation dataset *N* = 2,229	Validation dataset *N* = 248	*p*-value
Baseline characteristics			
Age, mean (SD), years	63.5 (13.1)	63.7 (12.2)	0.833
Median (IQR)	64.3 (19.6)	65.9 (17.0)	
Females, No. (%)	687 (30.8)	81 (32.7)	0.552
eGFR, mean (SD), ml/min 1.73 m^2^	70 (23)	70 (24)	0.954
Median (IQR)	71 (34)	72 (39)	
Arterial lactate, mean (SD), mmol/L	2.3 (2.2)	2.4 (2.2)	0.986
Median (IQR)	1.6 (1.7)	1.6 (1.8)	
Genetic syndrome, No. (%)	43 (1.9)	3 (1.2)	0.619
Bicuspid aortic valve, No. (%)	86 (3.9)	11 (4.4)	0.658
Iatrogenic dissection, No. (%)	59 (2.6)	7 (2.8)	0.871
Diabetes, No. (%)	112 (5.0)	13 (5.2)	0.882
Stroke, No. (%)	94 (4.2)	9 (3.6)	0.660
Pulmonary disease, No. (%)	173 (7.8)	31 (12.5)	0.010
Extracardiac arteriopathy, No. (%)	140 (6.3)	23 (9.3)	0.071
Poor mobility, No. (%)	85 (3.8)	11 (4.4)	0.630
Prior cardiac surgery, No. (%)	73 (3.3)	7 (2.8)	0.702
SPAP, mean (SD), mmHg			0.027
30–55	153 (6.9)	25 (10.1)	
>55	18 (0.8)	5 (2.0)	
LVEF ≤ 50%, No. (%)	524 (23.5)	46 (18.5)	0.078
Shock requiring inotropes, No. (%)	413 (18.5)	38 (15.3)	0.215
Preop. mechanical ventilation, No. (%)	254 (11.4)	20 (8.1))	0.113
Cardiopulmonary resuscitation,^a^ No. (%)	103 (4.6)	8 (3.2)	0.314
Preoperative malperfusion, No. (%)			
Cerebral	529 (23.7)	64 (25.8)	0.468
Spinal	49 (2.2)	6 (2.4)	0.823
Renal	211 (9.5)	28 (11.3)	0.356
Mesenteric	96 (4.3)	15 (6.0)	0.209
Peripheral	365 (16.4)	47 (19.0)	0.301
DeBakey type I dissection, No. (%)	1,941 (87.1)	217 (87.5)	0.795
Operative data			
Isolated ascend. aortic replacement, No. (%)	1,619 (72.6)	178 (71.8)	0.774
Partial/total arch replacement, No. (%)	401 (18.0)	45 (18.1)	0.952
Total aortic arch replacement, No. (%)	290 (13.0)	32 (12.9)	0.962
Frozen elephant trunk proc. No. (%)	126 (5.7)	15 (6.0)	0.799
Aortic root replacement No. (%)	610 (27.4)	70 (28.2)	0.774
Bentall–DeBono procedure, No. (%)	505 (22.7)	61 (24.6)	0.490
David procedure, No. (%)	71 (3.2)	7 (2.8)	0.756
Yacoub procedure, No. (%)	34 (1.5)	2 (0.8)	0.574
Concomitant coronary surgery, No. (%)	203 (9.1)	21 (8.5)	0.739
Concomitant mitral or tricuspid valve surgery, No. (%)	23 (1.0)	2 (0.8)	1.000

eGFR, estimated glomerular filtration rate according to the CKD-EPI equation; LVEF, left ventricular ejection fraction; SD, standard deviation; SPAP, systolic pulmonary artery pressure.

^a^ at arrival to the operating room or immediately before anesthesia induction.

### Outcomes in the derivation dataset

The rate of in-hospital mortality was 18.4%, stroke/global brain ischemia was 18.7%, dialysis was 15.3%, and acute heart failure was 16.4%. The rate of composite outcome was 41.2%. Ten-year mortality rate was 47.0%. Baseline variables associated with in-hospital mortality in the derivation dataset in univariable analysis are reported in Table 2.

**Table 2:** Baseline variables associated with in-hospital mortality in the derivation dataset in univariable analysis.

	Survivors *N* = 1,818	In-hospital deaths *N* = 411	*p*-value
Baseline characteristics			
Age, mean (SD), years	62.5 (13)	67.8 (12.7)	<0.0001
Median (IQR)	63.3 (19.6)	70.2 (18.4)	
Females, No. (%)	555 (30.5)	132 (32.1)	0.529
eGFR, mean (SD), ml/min 1.73 m^2^	73 (23)	59 (21)	<0.0001
Median (IQR)	74 (33)	58 (30)	
Arterial lactate, mean (SD), mmol/L	2.1 (1.8)	3.4 (3.2)	<0.0001
Median (IQR)	1.5 (1.4)	2.3 (2.9)	
Genetic syndrome, No. (%)	38 (2.1)	5 (1.2)	0.321
Bicuspid aortic valve, No. (%)	78 (4.3)	8 (2.0)	0.026
Iatrogenic dissection, No. (%)	40 (2.2)	19 (4.6)	0.006
Diabetes, No. (%)	82 (4.5)	30 (7.3)	0.019
Stroke, No. (%)	76 (4.2)	18 (4.4)	0.856
Pulmonary disease, No. (%)	136 (7.5)	37 (9.0)	0.298
Extracardiac arteriopathy, No. (%)	98 (5.4)	42 (10.2)	<0.0001
Poor mobility, No. (%)	57 (3.1)	28 (6.8)	<0.0001
Prior cardiac surgery, No. (%)	57 (3.1)	16 (3.9)	0.436
SPAP, mean (SD), mmHg			0.821
30–55	122 (6.7)	31 (7.5)	
>55	14 (0.8)	3 (0.3)	
LVEF ≤ 50%, No. (%)	365 (20.1)	159 (38.7)	<0.0001
Shock requiring inotropes, No. (%)	299 (16.4)	114 (27.7)	<0.0001
Preop. mechanical ventilation, No. (%)	164 (9.0)	90 (21.9)	<0.0001
Cardiopulmonary resuscitation^a^, No. (%)	55 (3.0)	45 (10.9)	<0.0001
Preoperative malperfusion, No. (%)			
Cerebral	391 (21.5)	138 (33.6)	<0.0001
Spinal	36 (2.0)	13 (3.2)	0.140
Renal	151 (8.3)	60 (14.6)	<0.0001
Mesenteric	58 (3.2)	38 (9.2)	<0.0001
Peripheral	273 (15.0)	92 (22.4)	<0.0001
DeBakey type I dissection, No. (%)	1,591 (87.5)	350 (85.2)	0.429

eGFR, estimated glomerular filtration rate according to the CKD-EPI equation; LVEF, left ventricular ejection fraction; SD, standard deviation; SPAP, systolic pulmonary artery pressure.

^a^ En route to the operating room or after anesthesia induction.

**Figure 2:**
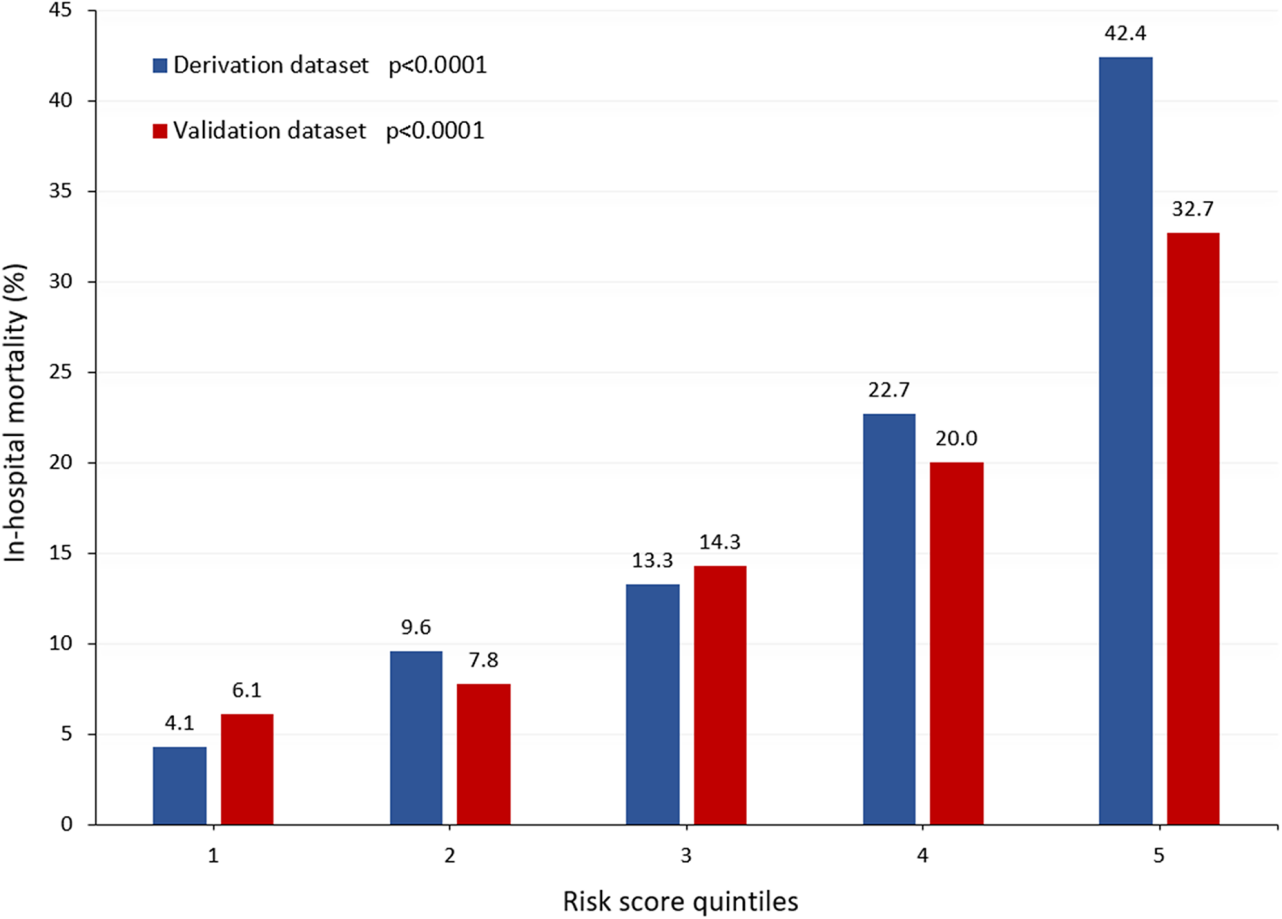
Rates of in-hospital mortality in quintiles of the risk score in the derivation and validation datasets (both datasets *p* < 0.0001).

**Figure 3:**
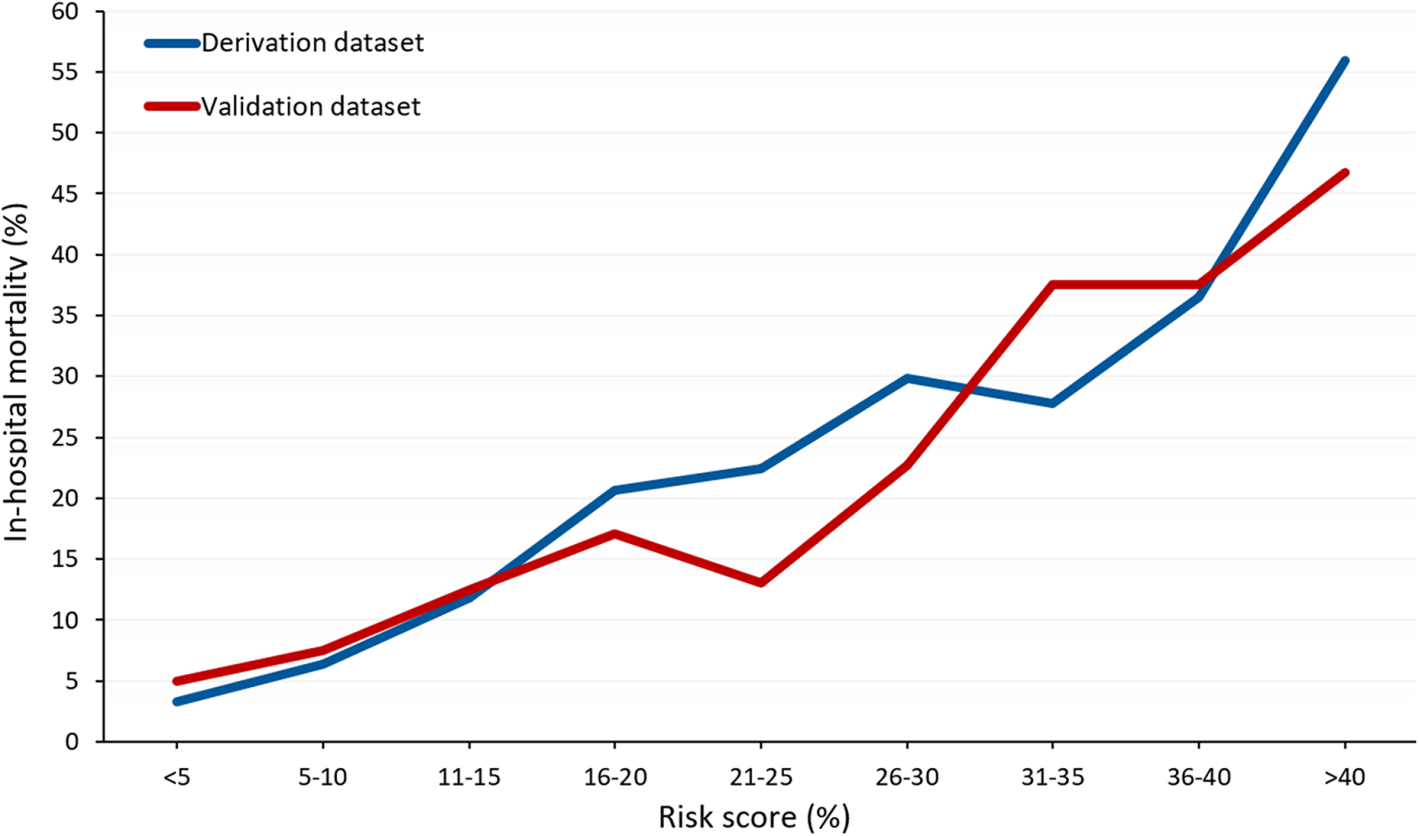
Observed rates of in-hospital mortality according to the risk score in the derivation and validation datasets (both datasets *p* < 0.0001).

Logistic regression identified the following variables associated with an increased risk of in-hospital mortality: age; eGFR; arterial lactate; iatrogenic dissection; left ventricular ejection fraction ≤50%; invasive mechanical ventilation; cardiopulmonary resuscitation immediately before surgery; and cerebral, mesenteric, and peripheral malperfusion (Table 3). This regression model had an AUC of 0.755 (95% CI, 0.729–0.780; Hosmer–Lemeshow test: *p* = 0.833, correlation 0.385, Brier score 0.128). The rates of in-hospital mortality significantly increased along quintiles of the risk score (*p* < 0.0001, Figure 2). Figure 3 summarizes the rate of in-hospital mortality according to the risk score.

**Table 3:** Baseline variables associated with in-hospital mortality in the derivation dataset in multivariable analysis.

	β coefficients	OR, 95% CI
Age	0.027466	1.028 (1.018–1.038)
eGFR	−0.017092	0.983 (0.978–0.989)
Arterial lactate	0.155482	1.168 (1.111–1.229)
Iatrogenic dissection, No. (%)	0.889842	2.435 (1.323–4.481)
LVEF ≤ 50%, No. (%)	0.628240	1.874 (1.449–2.425)
Invasive mechanical ventilation, No. (%)	0.612192	1.844 (1.332–2.554)
Cerebral malperfusion, No. (%)	0.291361	1.338 (1.030–1.738)
Mesenteric malperfusion, No. (%)	0.584877	1.795 (1.114–2.893)
Peripheral malperfusion, No. (%)	0.394077	1.483 (1.098–2.003)
Cardiopulmonary resuscitation^a^, No. (%)	0.519416	1.681 (1.046–2.701)
Constant βo	−3.066941	

CI, confidence interval; eGFR, estimated glomerular filtration rate according to the CKD-EPI equation; LVEF, left ventricular ejection fraction; OR, odds ratio.

^a^ En route to the operating room or after anesthesia induction.

**Figure 4:**
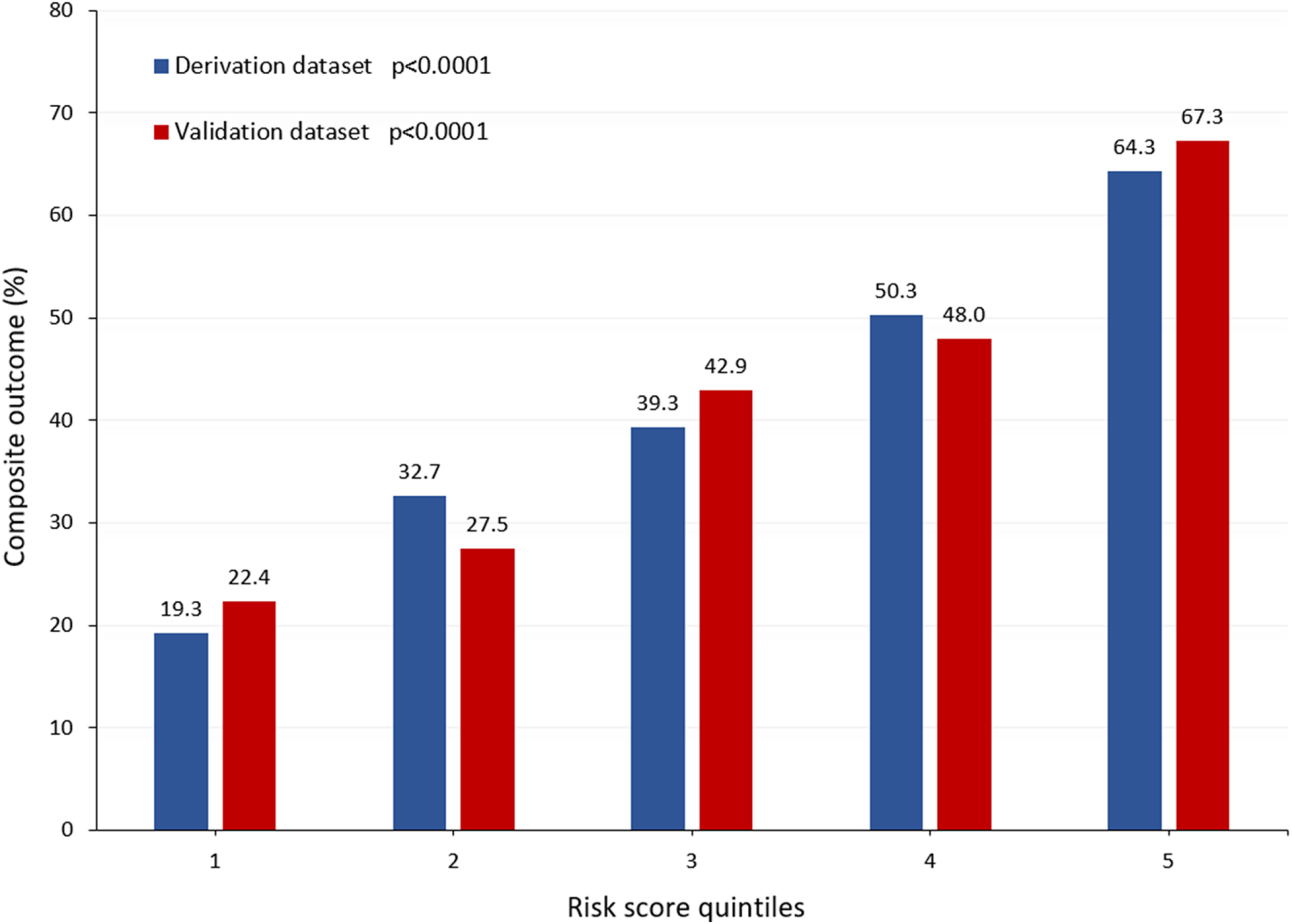
Rates of composite outcome (in-hospital death, stroke/global brain ischemia, dialysis, and acute heart failure during the index hospitalization) in quintiles of the risk score in the derivation and validation datasets (both datasets *p* < 0.0001).

The estimated risk score was associated with an increased risk of composite outcome (AUC, 0.689, 95% CI, 0.667–0.711) as well as postoperative stroke/global brain ischemia (AUC, 0.617, 95% CI, 0.588–0.646), dialysis (AUC, 0.657, 95% CI, 0.627–0.688), and acute heart failure (AUC, 0.661, 95% CI, 0.631–0.692). Figure 4 summarizes the rates of composite outcome in quintiles of the risk score (*p* < 0.0001).

**Figure 5:**
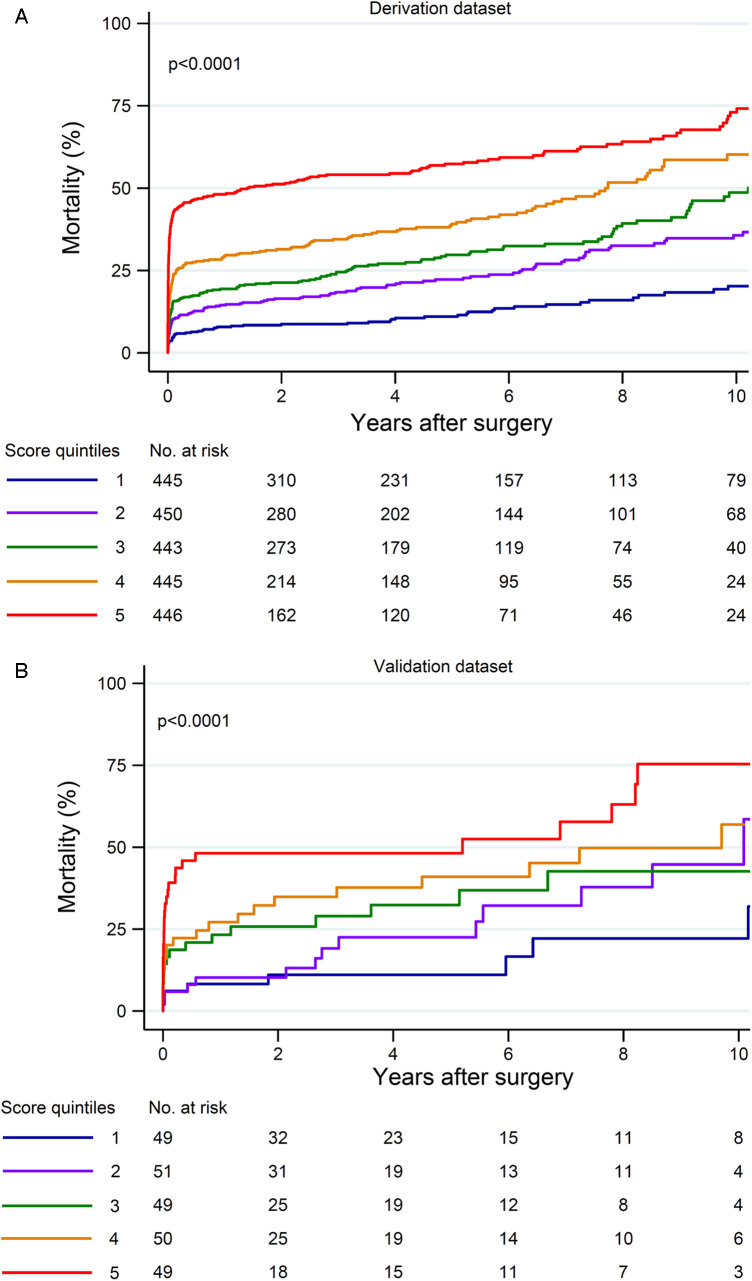
Ten-year mortality in quintiles of the risk score in the derivation (**A**) and validation (**B**) datasets (both datasets *p* < 0.0001).

The estimated risk score was predictive of late mortality (HR, 1.035, 95% CI, 1.031–1.038; Harrell's C 0.702; Somer's D 0.403, Brier score 0.234), also when hospital deaths were excluded from the analysis (HR, 1.024, 95% CI, 1.018–1.031; Harrell's C 0.630; Somer's D 0.261, Brier score 0.170). Late mortality rates according to quintiles of the risk score are shown in Figure 5A (Log-rank test: *p* < 0.0001).

### Outcomes in the validation dataset

In the validation dataset, the rate of in-hospital mortality rate was 16.1%. The rate of postoperative of stroke/global brain ischemia was 17.3%, dialysis 17.7%, and acute heart failure 14.5%. The rate of composite outcome was 41.5%. Ten-year mortality rate was 49.1%.

**Figure 6:**
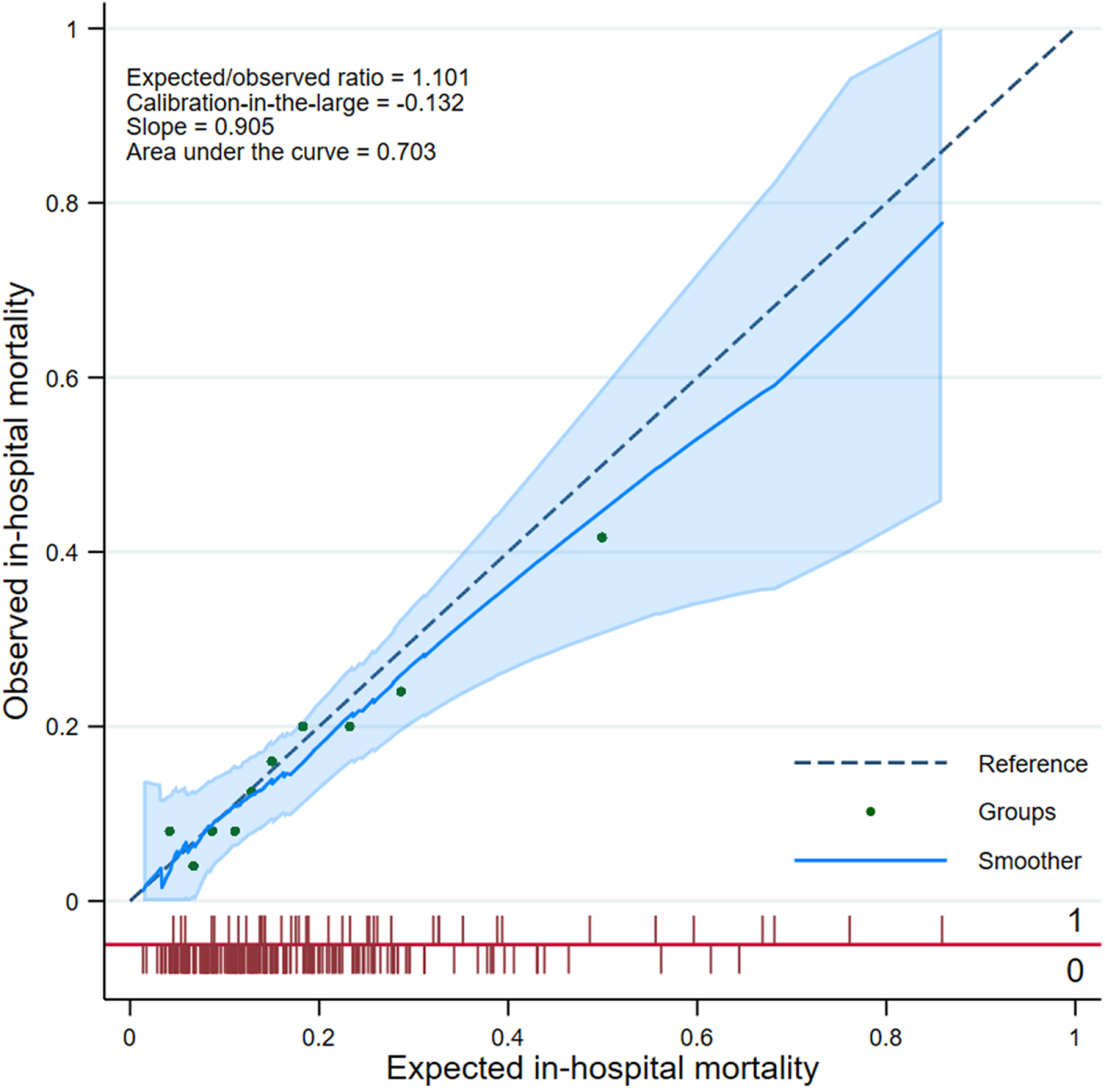
Calibration plot of the risk score in the validation dataset.

The estimated risk score was predictive of in-hospital mortality (AUC, 0.703, 95% CI, 0.613–0.793; Hosmer–Lemeshow test: *p* = 0.974). While the probability of in-hospital mortality was 0.161, that of prediction was 0.178, with a correlation of 0.326, a Brier score of 0.121, and a slope of 0.905 (Figure 6).

The rates of in-hospital mortality increased along quintiles of the risk score (*p* < 0.0001, Figure 2). This risk score was associated with an increased risk of composite outcome (AUC, 0.682, 95% CI, 0.614–0.749), postoperative stroke/global brain ischemia (AUC, 0.652, 95% CI, 0.565–0.739), and acute heart failure (AUC, 0.639, 95% CI, 0.533–0.746). This score tended to also predict the risk of postoperative dialysis (AUC, 0.589, 95% CI, 0.498–0.680). Figure 4 summarizes the rates of composite outcome in quintiles of the risk score (*p* < 0.0001).

The estimated risk score was predictive of late mortality (HR, 1.036, 95% CI, 1.024–1.049; Harrell's C 0.678; Somer's D 0.356, Brier score 0.244) also when hospital deaths were excluded from the analysis (HR, 1.027, 95% CI, 1.005–1.050; Harrell's C 0.649; Somer's D 0.298, Brier score 0.183). Late mortality rates according to quintiles of this score are shown in Figure 5B (Log-rank test: *p* < 0.0001).

### Independent predictors of in-hospital mortality in the entire dataset

In the entire dataset, the rate of in-hospital mortality rate was 18.2.1%. Logistic regression confirmed the findings observed in the derivation dataset with similar risk estimates of the independent variables (Table 4). The AUC of the probabilities of this regression model was comparable to that of the probabilities estimated from the derivation dataset (0.751, 95% CI, 0.726–0.776 vs. 0.750, 95% CI, 0.725–0.775, *p* = 0.266). The estimated root mean squared error ranged from −351 to −367, the pseudo-R^2^ from 0.107 to 0.148, the mean absolute errors from 0.257 to 0.268, and mean likelihood from −843 to −827 in fivefold.

**Table 4:** Baseline variables associated with in-hospital mortality in the entire dataset in multivariable analysis.

	β coefficients	OR (95%CI)
Age	0.025515	1.026 (1.016–1.036)
eGFR	−0.01569	0.984 (0.979–0.990)
Arterial lactate	0.153442	1.166 (1.111–1.223)
Iatrogenic dissection, No. (%)	0.896671	2.451 (1.380–4.356)
LVEF ≤ 50%, No. (%)	0.639263	1.895 (1.482–2.423)
Invasive mechanical ventilation, No. (%)	0.637766	1.892 (1.384–2.586)
Cerebral malperfusion, No. (%)	0.329356	1.390 (1.086–1.780)
Mesenteric malperfusion, No. (%)	0.583768	1.793 (1.146–2.806)
Peripheral malperfusion, No. (%)	0.407691	1.503 (1.132–1.997)
Cardiopulmonary resuscitation^a^, No. (%)	0.655331	1.895 (1.482–2.423)
Constant βo	−3.064043	

CI, confidence interval; eGFR, estimated glomerular filtration rate according to the CKD-EPI equation; LVEF, left ventricular ejection fraction; OR, odds ratio.

^a^ En route to the operating room or after anesthesia induction.

## Discussion

This study confirmed that current rates of early mortality, major adverse complications, and 10-year mortality after surgery for TAAD are still high. The identification of determinants of poor outcome in these patients is of utmost importance to stratify their operative risk, because it may guide clinicians in the decision-making process by avoiding extensive surgery or even planning endovascular/hybrid procedures in TAAD patients at highest operative risk (6). Therefore, the development of risk scoring methods is not a mere mathematical exercise, but an important means for a more in-depth grading of the severity of clinical conditions of TAAD patients. Indeed, a reliable clinical risk score would be a valuable tool of risk adjustment in the evaluation of surgical and anesthesiological treatment methods, particularly in TAAD patients in whom these methods are not yet standardized.

Our analysis showed that aortic dissection–related conditions requiring invasive mechanical ventilation and cardiopulmonary resuscitation en route to the operating room or after anesthesia induction are the main determinants of poor outcome along with depressed left ventricular ejection fraction and cerebral, mesenteric, and peripheral malperfusion. In this series, iatrogenic aortic dissection was associated with a rather high risk in-hospital mortality. Such an increased mortality risk may be due to underlying cardiac and extracardiac comorbidities in patients undergoing invasive cardiovascular procedures as well as prolonged myocardial ischemia in iatrogenic TAAD occurring immediately after cardiac surgery (12, 13). Advanced age is recognized as a major risk factor in cardiovascular surgery, particularly in acute aortic syndromes (14–17) as confirmed in the present analysis. Most of these risk factors were identified as independent predictors of early mortality after surgery for TAAD also by the UK National Adult Cardiac Surgical Audit (2) and the German Registry of Acute Aortic Dissection Type A (GERAADA) (7) investigators. The regression models of these two large studies estimated risk scores with AUCs of 0.63 and 0.73, respectively. However, the lack of information on the regression constant β coefficient makes difficult to retrospectively estimate these risk scores in large series.

The present study showed that, when preoperative eGFR and arterial lactate were included in the logistic regression model, the estimated probabilities of in-hospital mortality had a rather large AUC with a low Brier score. In fact, after excluding these risk factors from the regression model of the derivation dataset, the AUC of the probabilities of in-hospital mortality was 0.708 (95% CI, 0.680–0.736) with a Brier score of 0.135. Recent studies showed that biomarkers may be helpful in stratifying the operative risk of TAAD patients (6, 18, 19). In particular, preoperative levels of creatinine and arterial lactate have been shown to be significant predictors of early postoperative mortality (6, 18, 19). The prognostic impact of eGFR on adverse events after cardiac surgery has been largely demonstrated (20), while the value of arterial lactate in predicting the outcome of critically ill cardiac surgery patients is rather novel (6, 18, 19, 21). A recent study by Nappi et al. (19) showed that preoperative arterial lactate was an independent risk factor for early postoperative mortality (OR, 1.378, 95% CI, 1.176–1.616) along with eGFR (OR, 0.978, 95% CI, 0.991–0.997) after surgery for TAAD. The authors identified a cutoff of 2.6 mmol/L for arterial lactate, which was associated with a significantly higher rate of early mortality (44.6% vs. 17.8%, OR, 4.07, 95% CI, 2.43–7.78) (19). Ghoreishi et al. (18) identified preoperative lactic acid level (OR, 1.39, 95%CI, 1.45–20.0) along with serum creatinine and increased liver enzymes (aspartate aminotransferase >50 mmol/L, alanine aminotransferase >55 mmol/L, bilirubin >1.2 mg/dl) as independent risk factors for early mortality in patients who underwent surgical repair for TAAD. These findings suggest that including objective parameters of preoperative renal failure and reduced oxygen delivery/end-organ ischemic injury may provide additional prognostic information on the clinical status of these critically ill patients. Interestingly, these biomarkers may provide prognostic information also to estimate the risk of major postoperative complications as demonstrated by the present findings.

The present study has several limitations that must be considered. First, the retrospective nature of this registry is the main limitation of this study. Second, complete collection of baseline characteristics of TAAD patients might have been difficult in the emergency setting, particularly for those undergoing expedite surgery. Third, we gathered data on myocardial malperfusion, but we did not consider this variable in the present analysis because retrospective data on myocardial infarction in TAAD patients with severe hemodynamic instability can be biased. In the derivation dataset, logistic regression including myocardial malperfusion showed that it was an independent predictor of in-hospital mortality (OR, 1.644, 95% CI, 1.207–2.238), but it did not increase the predictive ability of the regression model (AUC, 0.758, 95% CI, 0.732–0.783; Brier score 0.128). Fourth, similar to the GERAADA study, operative variables were not considered in this analysis because we sought to identify baseline risk factors which may be useful for risk adjustment in the evaluation of different treatment strategies for acute TAAD. Fifth, interinstitutional differences of referral pathways, patients’ operative risk, and treatment strategies might impact the present findings. Multilevel mixed-effects logistic regression addressing the cluster effect of participating hospitals confirmed that all baseline variables herein identified were independent predictors of in-hospital mortality, but the AUC of the estimated probabilities was larger (AUC, 0.777, 95% CI, 0.752–0.801; Brier score 0.124). However, when validating the probabilities estimated by this type of regression, we believe that there is no appropriate way to consider the effect of these clusters in other study populations. Sixth, we do not have complete data on the main causes of death of these patients for a more in-depth analysis of the effect of baseline and operative variables on fatal adverse complications. Seventh, the exclusion of patients without data on preoperative arterial lactate level might be considered a potential weakness of this study. However, we have previously observed that including preoperative arterial lactate levels improved the predictive accuracy of the regression model for prediction of in-hospital mortality (22). Therefore, we chose to include only patients with data on preoperative arterial lactate in this analysis. Eight, the methodology of splitting the study cohort into a derivation and validation datasets is largely adopted for the development and internal validation of risk scoring methods. The k-fold cross-validation in the entire study cohort is adopted for this purpose as well. This method was applied in this study to validate the predictive accuracy of the estimated probabilities. Finally, internal validation of any risk score has some limitations. Therefore, external validation of the present risk score is awaited to confirm its predictive accuracy.

The strengths of the study are the large size of the study population, the availability of data on preoperative serum creatinine and arterial lactate, the heterogeneity of referral pathways, and level of perioperative treatment as well as the early mortality rate, which were comparable to recent large national series (2, 3). These aspects may make the findings of this study generalizable to other TAAD study populations.

## Conclusions

The present analysis showed that age, eGFR, arterial lactate, depressed left ventricular function, and TAAD-related clinical variables are predictive of in-hospital mortality and other major postoperative adverse events. These risk factors may be valuable components for risk adjustment in the evaluation of surgical and anesthesiological strategies aiming to improve the results of surgery for TAAD. External validation of the present risk score is awaited to confirm its predictive accuracy.

## Data Availability

The datasets presented in this article are not readily available because the authors do not have the permission to publicly share these data. Requests to access the datasets should be directed to faustobiancari@yahoo.it.
